# Singleton Merten Syndrome: A Rare Cause of Early Onset Aortic Stenosis

**DOI:** 10.1155/2017/8197954

**Published:** 2017-02-21

**Authors:** Harshavardhan Ghadiam, Sudhir Mungee

**Affiliations:** ^1^Department of Medicine, University of Illinois College of Medicine at Peoria, Peoria, IL, USA; ^2^Division of Cardiology, University of Illinois College of Medicine at Peoria, Peoria, IL, USA

## Abstract

Singleton Merten syndrome (SMS) is a rare autosomal dominant genetic disorder with variable expression. Its characteristic features include abnormal aortic calcification, abnormal ossification of extremities, and dental anomalies. We present a young man with dyspnea who was noted to have aortic stenosis in the background of glaucoma, psoriasis, dental anomalies, hand and foot deformities, Achilles tendinitis, osteopenia, and nephrolithiasis. The conglomeration of features led to the diagnosis of SMS. His mother had a very similar phenotype.

## 1. Introduction

SMS is an autosomal dominant disorder with variable expression [1]. It is associated with abnormal calcification in vascular and connective tissues causing aortic calcification and aortic valve stenosis [1]. The pathophysiology of abnormal calcification is unknown but is likely linked to gain-of-function IFIH1 mutation [2]. We had a young male patient who presented for dyspnea and palpitations and was noted to have mild aortic stenosis.

## 2. Case Report

**Figure 1:**
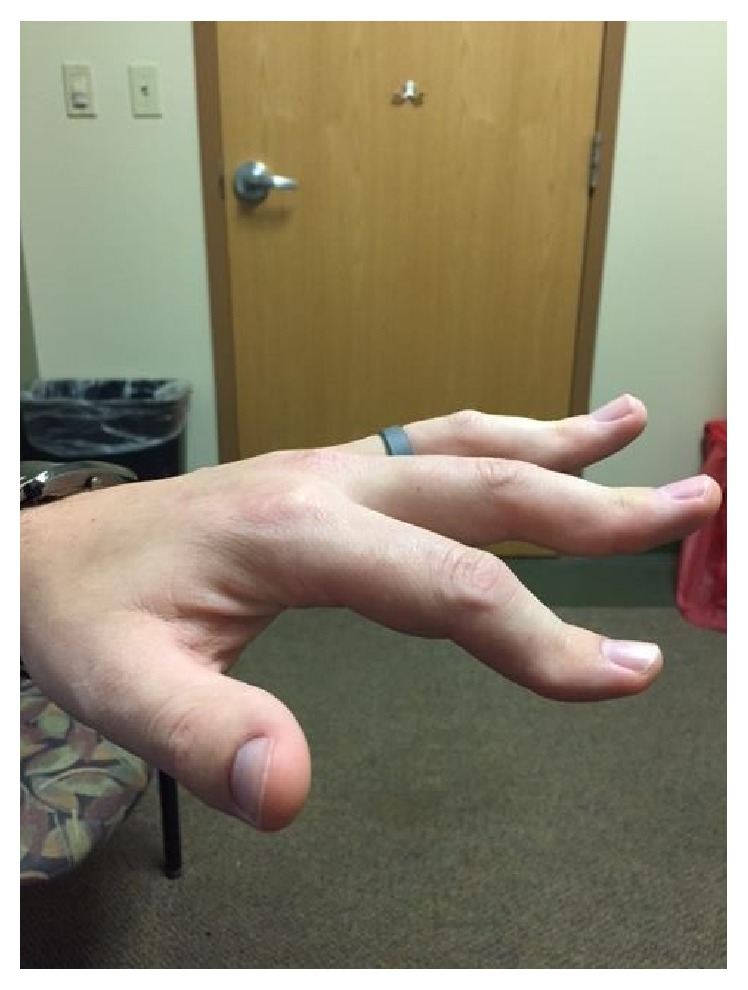
Hand deformities.

**Figure 2:**
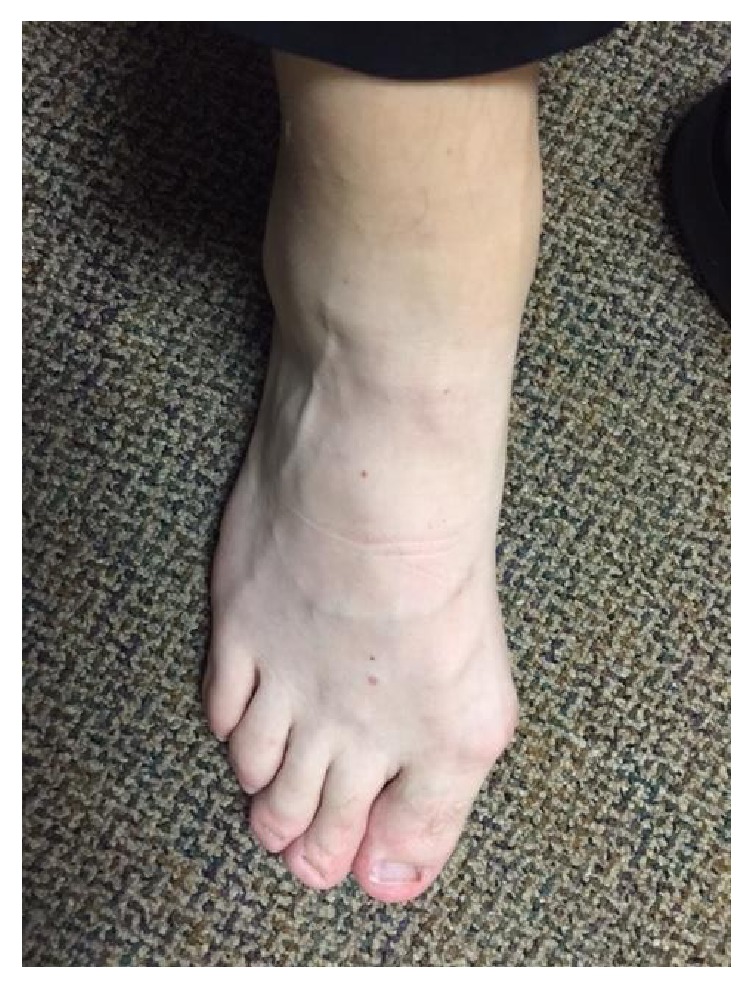
Hallux valgus.

A 30-year-old male with a past medical history of early onset glaucoma, multiple trabeculectomies, delayed eruption of permanent dentition, progressive hand deformities (Figure 1), psoriasis, recurrent nephrolithiasis, Achilles tendinitis, tendon rupture, foot deformities (Figure 2), and hypoplastic toe nails presented for evaluation of dyspnea on exertion and palpitations. Symptoms have been ongoing for three months with gradual worsening. His family history was significant for premature coronary artery disease in his mother and aortic stenosis with aortic valve replacement when she was 31 years old.

**Figure 3:**
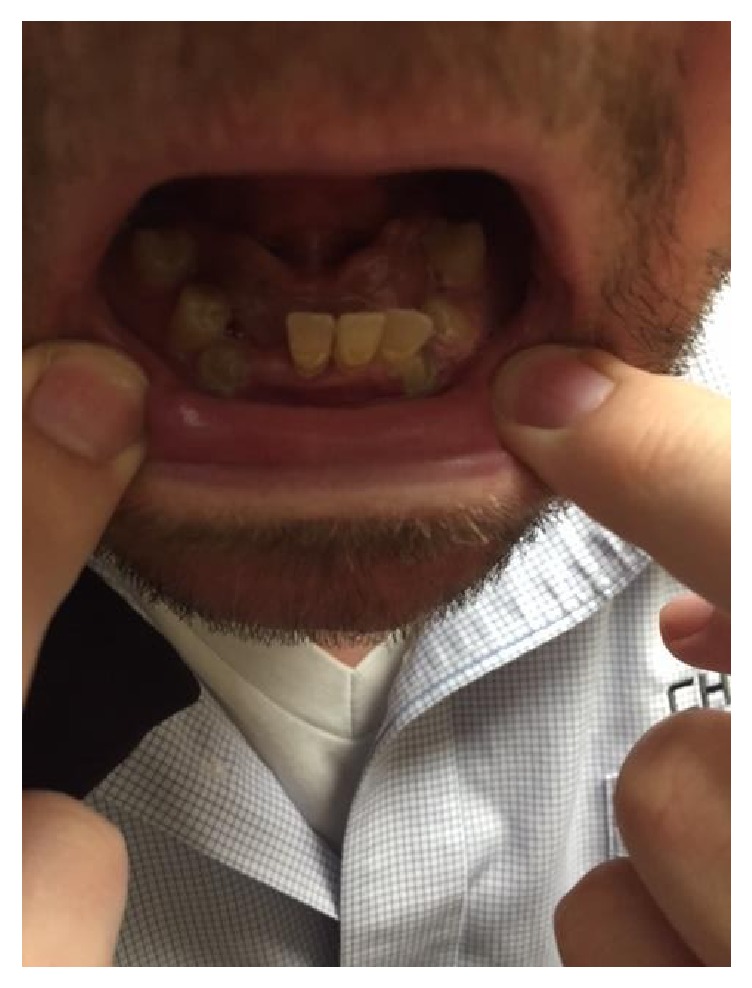
Dysplastic permanent teeth.

Physical examination showed grade 3/6 systolic murmur in the left second intercostal space, hallux valgus deformities in the feet (Figure 2), and dysplastic dentition (Figure 3).

**Figure 4:**
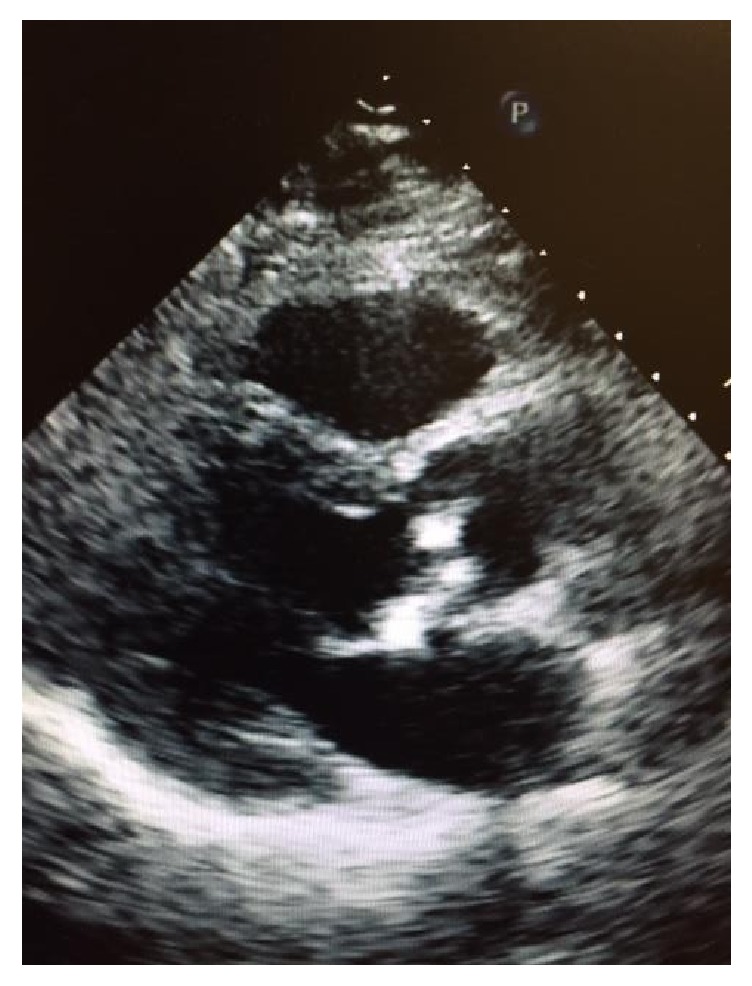
Aortic stenosis with calcified leaflets.

The patient underwent an echocardiogram which revealed moderate mitral annular calcification, mild aortic stenosis (Figure 4) with partial fusion of noncoronary and left coronary cusps, and calcified leaflets, with a peak gradient of 24.4 mm Hg and mean gradient of 13 mm Hg and peak velocity of 2.47 m/s. Thirty-day event recorder was unremarkable. Imaging revealed minimal hyperostosis at triceps insertion on the ulna. It also revealed right hallux valgus deformity and peritendinous calcification of bilateral patellar tendons. CT chest revealed minimal calcification of aortic arch and abdominal aorta, calcification of aortic valve, and ductus arteriosus remnant. Genetic testing revealed autosomal dominant IFIH1 mutation.

Repeat echocardiogram in one year showed moderate aortic stenosis with peak velocity of 3.16 m/s, peak gradient of 40 mmHg and mean gradient of 20 mmHg, and aortic valve of 0.98 cm^2^.

## 3. Discussion

Our patient had features similar to Singleton Merten syndrome including glaucoma, dysplastic permanent teeth, multiple tendon rupture, and extensive vascular calcification. Singleton Merten syndrome is an autosomal dominant disorder with variable expression. His mother had a similar phenotype.

SMS is a rare disease, with very few cases reported in the literature. Common features include abnormal aortic calcification, abnormal ossification of extremities, and dental anomalies [3, 4]. Other associated features are glaucoma, psoriasis [4], tendinitis, and osteoporosis [1].

The pathophysiology of the disease is unclear in regard to the abnormal calcification of vascular and connective tissues with a possible link to abnormal calcium metabolism [1, 5]. Our patient had normal laboratory evaluation for calcium metabolism. Genetic testing in subjects with SMS has revealed a gain-of-function IFIH1 mutation likely causing premature arterial calcification and dental inflammation [2].

The patient is currently followed up by a multidisciplinary team and is being monitored for the progression of aortic stenosis.

Although this disease is not unique, not many cases have been described before. Due to its rarity, we hope this case would help in understanding this rare and complex disease.
